# Molecular mechanisms underlying glucose-dependent insulinotropic polypeptide secretion in human duodenal organoids

**DOI:** 10.1007/s00125-024-06293-3

**Published:** 2024-10-23

**Authors:** Nunzio Guccio, Constanza Alcaino, Emily L. Miedzybrodzka, Marta Santos-Hernández, Christopher A. Smith, Adam Davison, Rula Bany Bakar, Richard G. Kay, Frank Reimann, Fiona M. Gribble

**Affiliations:** 1 Institute of Metabolic Science, Addenbrooke’s Hospital, University of Cambridge, Cambridge, UK; 2 Present Address: Centre for Regenerative Medicine, Institute for Regeneration and Repair, University of Edinburgh, Edinburgh, UK

**Keywords:** CASR, GIP, GPR142, Organoid, SGLT1

## Abstract

**Aims/hypothesis:**

Glucose-dependent insulinotropic polypeptide (GIP) is an incretin hormone secreted by enteroendocrine K cells in the proximal small intestine. This study aimed to explore the function of human K cells at the molecular and cellular levels.

**Methods:**

CRISPR-Cas9 homology-directed repair was used to insert transgenes encoding a yellow fluorescent protein (Venus) or an Epac-based cAMP sensor (Epac-S-H187) in the GIP locus in human duodenal-derived organoids. Fluorescently labelled K cells were purified by FACS for RNA-seq and peptidomic analysis. GIP reporter organoids were employed for GIP secretion assays, live-cell imaging of Ca^2+^ using Fura-2 and cAMP using Epac-S-H187, and basic electrophysiological characterisation. The G protein-coupled receptor genes *GPR142* and *CASR* were knocked out to evaluate roles in amino acid sensing.

**Results:**

RNA-seq of human duodenal K cells revealed enrichment of several G protein-coupled receptors involved in nutrient sensing, including *FFAR1*, *GPBAR1*, *GPR119*, *CASR* and *GPR142*. Glucose induced action potential firing and cytosolic Ca^2+^ elevation and caused a 1.8-fold increase in GIP secretion, which was inhibited by the sodium glucose co-transporter 1/2 (SGLT1/2) blocker sotagliflozin. Activation of the long-chain fatty acid receptor free fatty acid receptor 1 (FFAR1) induced a 2.7-fold increase in GIP secretion, while tryptophan and phenylalanine stimulated secretion by 2.8- and 2.1-fold, respectively. While *CASR* knockout blunted intracellular Ca^2+^ responses, a *CASR*/*GPR142* double knockout was needed to reduce GIP secretory responses to aromatic amino acids.

**Conclusions/interpretation:**

The newly generated human organoid K cell model enables transcriptomic and functional characterisation of nutrient-sensing pathways involved in human GIP secretion. Both calcium-sensing receptor (CASR) and G protein-coupled receptor 142 (GPR142) contribute to protein-stimulated GIP secretion. This model will be further used to identify potential targets for modulation of native GIP secretion in diabetes and obesity.

**Graphical Abstract:**

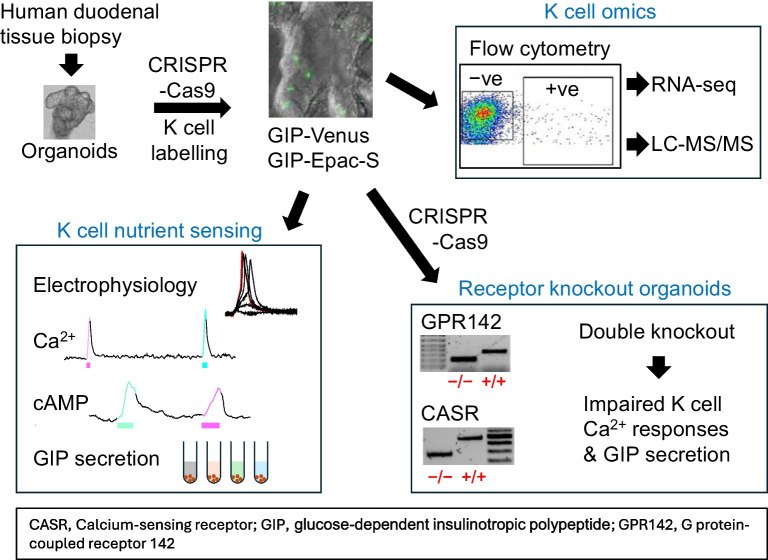

**Supplementary Information:**

The online version contains peer-reviewed but unedited supplementary material available at 10.1007/s00125-024-06293-3.


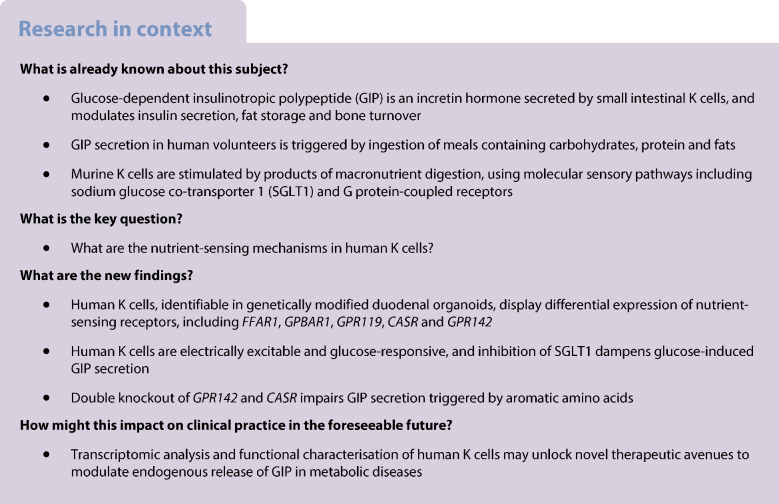


## Introduction

Glucose-dependent insulinotropic polypeptide (GIP) is secreted by enteroendocrine K cells in the duodenum and plays a major role in the physiological incretin effect, alongside its sister incretin glucagon-like peptide 1 (GLP-1) [1]. Whereas GLP-1 forms the basis for many glucose-lowering and anti-obesity drugs such as liraglutide and semaglutide [2, 3], GIP-based therapeutics have lagged behind due to early concerns that GIP had only weak activity in the context of type 2 diabetes [4]. However, GLP-1 receptor (GLP1R)/GIP receptor (GIPR) dual agonists have now arrived on the market and induce more weight loss and better glucose control than many current drugs targeting GLP1R alone [3, 5], reigniting interest in human GIP physiology.

Plasma GIP concentrations in humans rise rapidly following oral glucose ingestion and act as an early signal to pancreatic islets that glucose is being absorbed [6]. Robust GIP responses are also observed after ingestion of meals containing carbohydrates, fats or proteins [7, 8]. At the cellular and molecular levels, characterisation of human enteroendocrine cells (EECs), including GIP-secreting K cells, is currently lacking because of an absence of in vitro models. In mice, glucose-triggered GIP secretion has been attributed to sodium glucose co-transporter 1 (SGLT1) activity, and a variety of nutrient-responsive G protein-coupled receptors (GPCRs) such as the long-chain free fatty acid receptor 1 (FFAR1) have been implicated in GIP responses to different macronutrients [1]. Understanding how human K cells sense nutrients and other stimuli is key to explaining postprandial physiology and unlocking the therapeutic potential of K cells as drug targets, potentially in combination with a dipeptidyl peptidase-4 (DPP4) inhibitor to prolong the plasma half-life of GIP. The generation of organoids from intestinal biopsies, which can be genetically modified by CRISPR-Cas9, has allowed these questions to be approached using reproducible in vitro human models [9].

The aims of this study were to label and functionally characterise human K cells. K cells in human duodenal organoids were genetically engineered using CRISPR-Cas9 to express either the yellow fluorescent protein (YFP) Venus or the fluorescence resonance energy transfer (FRET)-based cAMP sensor Epac-S-H187. These reporter organoid lines were used to characterise K cells by RNA-seq, live-cell calcium and cAMP imaging, electrophysiology and in vitro GIP secretion assays. CRISPR-Cas9 was used to knockout (KO) two GPCRs, *GPR142* and *CASR*, to evaluate their contributions to amino acid (AA) detection by human K cells.

## Methods

### Human organoid culture and maintenance

Human duodenal organoids were generated from anonymous surgical samples from Addenbrooke’s Hospital Tissue Bank (Cambridge, UK), under ethical approval by the East of England–Cambridge Central Research Ethics Committee (no. 09/H0308/24). Duodenal organoids were generated and maintained as previously described [10, 11]. Cultures were fed twice weekly and passaged every 7–14 days. For passaging, one mature dome of organoids was incubated in Gibco TrypLE (Thermo Fisher, Bishops Stortsford, UK) at 37°C for 6–8 min, mechanically sheared using a pipette and seeded into pre-warmed 12-well plates. To promote K cell differentiation, EGF was removed 2–3 days after seeding (while retaining IGF1 and FGF2, called IF medium [12]) for 10–14 days. Cultures were then supplemented with 10 μmol/l Notch inhibitor DAPT (Generon, Slough, UK) and 100 nmol/l MEK inhibitor PD0325901 (Merck, Gillingham, UK) overnight. Subsequently, domes were cultured in IF medium until optimal K cell differentiation was achieved (3–7 days later).

### Generation of GIP-Venus human duodenal organoids

CRISPR-associated protein 9 (Cas9) induced homology-directed repair (HDR) was used to knock in either Venus or the FRET cAMP reporter Epac-S-H187 [13] following a picornavirus peptide-2A (P2A) sequence to enable bicistronic expression under control of the GIP promoter. A CRISPR site (GGTCAGAGTCACCGAGACCT**GGG**)—the protospacer adjacent motif (PAM) is highlighted in bold—in exon 6 was targeted. Single guide RNA (sgRNA)-Cas9 and donor plasmids were generated, purified and prepared for electroporation, as described previously [10, 11] and in the electronic supplementary material (ESM) Methods. Successful recombinants were enriched by adding G418 (0.5 mg/ml) to media 3–7 days after electroporation. Surviving organoids were manually picked and seeded individually in basement membrane extract (BME) domes. DNA was extracted from each organoid using QuickExtract DNA Extraction Solution (Lucigen Corporation, USA) and successful integration was assessed by PCR genotyping and confirmed by Sanger sequencing (Source Bioscience, Cambridge, UK).

### Generation of *GPR142* and *CASR* KO GIP-Venus human duodenal organoid lines

CRIPSR-Cas9 non-homologous end joining (NHEJ) was used to KO *GPR142* and *CASR*. *GPR142* guides (TGACCAGGAACACGCCACAA**GGG**, GGTAGAGCATGACGAAGACC**CGG**) targeted transmembrane domains 5 and 6 (exon 4); *CASR* guides (GGACACGGTTGGTTTTCACC**AGG**, ATCTTCATCACGTGCCACGA**GGG**) targeted transmembrane domain 3 and extracellular loop 2 (exon 7). Guide sequences were cloned into plasmids for electroporation as above. Genomic DNA was extracted and screened by PCR amplification to identify clones with biallelic deletions.

### cDNA library preparation and RNA-seq

RNA extraction and sequencing were performed after FACS as previously described [10, 11] (see ESM Methods for further details). Gene expression is presented in transcripts per million. Differential expression was calculated using the Wald test (default in DESeq2 [14] version 1.42.0), comparing GIP-Venus positive vs negative. RNA-seq data are deposited in the National Center for Biotechnology Information–Gene Expression Omnibus (NCBI GEO) repository (GSE271017).

### Peptidomic analysis

Peptide extraction and analysis of FACS-sorted cells were performed by LC-MS/MS as previously described [10, 11] (see ESM Methods for further details). Data have been deposited at the ProteomeXchange Consortium via the PRIDE partner repository (PXD052659).

### Secretion assays

Differentiated organoids were liberated from domes using ice-cold advanced DMEM/F-12 medium (ADF) (Gibco, Thermo Fisher, Bishops Stortsford, UK) and centrifuged at 400 *g* for 4 min. Organoids were washed twice for 30 min at 37°C with saline buffer (composition defined below) supplemented with 1 mmol/l glucose and 0.1% BSA. Intact 3D organoids were distributed into V-bottom 96-well plates and incubated in duplicates or triplicates with test reagents dissolved in saline buffer for 2 h at 37°C. Subsequently, plates were centrifuged at 2000 *g* for 5 min at 4°C, and supernatants were snap-frozen prior to analysis. Total GIP levels were measured by electrochemiluminescence (ECL) immunoassay (MesoScale Discovery, Rockville, MD, USA, no. K1515SK).

### Calcium and cAMP imaging

Calcium imaging was performed as previously described after loading with the acetoxymethyl ester of Fura-2 (Fura-2-AM) [10, 11, 15] (see ESM Methods for further details). cAMP-dependent FRET imaging was performed on GIP-Epac-S-H187 organoids as described previously [16] (see ESM Methods for further details).

### Electrophysiology

Electrophysiological recordings were performed on fluorescently labelled K cells as previously described [10, 11] (see ESM Methods for further details).

### Buffers

Saline buffer for imaging, electrophysiology and secretion contained (in mmol/l): 138 NaCl, 4.5 KCl, 4.2 NaHCO_3_, 1.2 NaH_2_PO_4_, 2.6 CaCl_2_, 1.2 MgCl_2_, 10 HEPES; adjusted to pH 7.4 with NaOH. Internal pipette solution for perforated patch recordings contained (in mmol/l): 76 K_2_SO_4_, 10 NaCl, 10 KCl, 10 HEPES, 55 sucrose, 1 MgCl_2_; adjusted to pH 7.2 with KOH. Amphotericin-B was dissolved in DMSO and added fresh to pipette solution at a final concentration of 200 µg/ml on the day of recording.

### Randomisation

Most experiments outlined in this article were not randomised. If agonists were used sequentially during imaging or electrophysiological experiments the order of application was varied to minimise sequential effects.

### Masking/blinding

For most experiments blinding was not possible. Exceptions are secretion experiments and peptidomic analysis, in which the staff analysing hormonal contents were blinded to the test conditions and sample identity.

### Inclusion and exclusion criteria

All data collected were included in the analysis with the exception of cells not responding to positive controls in the live-cell imaging experiments, as stated in the Data analysis section.

### Data analysis

Statistical tests were performed using GraphPad Prism (version 10, GraphPad Software, USA), DESeq2 (RNA-seq) or R (version 12, R Core Team, Austria), as indicated in individual figure legends. Cells in imaging experiments were included for analysis if they showed a response to the positive control: KCl (for Ca^2+^) or forskolin/3-isobutyl-1-methylxanthine (IBMX) (for cAMP). Cells were classified as ‘responders’ if the *z* score was >3 for at least two consecutive timepoints during perfusion of test substance; *z* score = [(*F*_*t*_ − mean *F*_b_)/SD *F*_b_], where *F*_*t*_ is the 340/380 or cyan/yellow fluorescent protein (CFP/YFP) ratio at time *t*, mean *F*_b_ is the mean basal fluorescence ratio calculated from 60 s of datapoints prior to test addition and SD *F*_b_ is the SD of *F*_b_ during the basal 60 s recording period.

## Results

### Generation of GIP-Venus and GIP-Epac-S-H187 human duodenal reporter lines

**Fig. 1:**
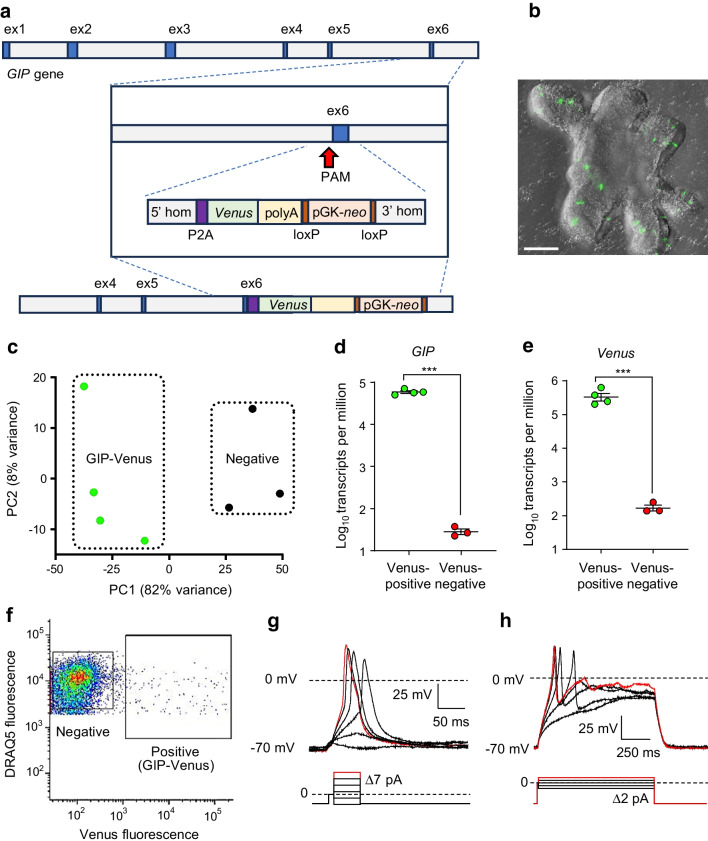
Human GIP-Venus duodenal organoids are electrically excitable. (**a**) Schematic representing the knockin strategy to insert the *Venus* transgene in exon 6 of the *GIP* gene using CRISPR-Cas9 HDR, allowing for bicistronic expression of the *Venus* gene under the GIP promoter. (**b**) Representative image of a GIP-Venus human organoid generated using a Celldiscoverer 7 system, equipped with a Plan-Apochromat ×5 objective (numerical aperture [NA] 0.35) coupled with a ×2 tube lens and an Axiocam 506 CCD camera (Zeiss, Cambridge, UK). The Venus signal was imaged using a 470 nm light-emitting diode (LED) light source and 524/50 emission filter (depicted in green), and phase gradient contrast images using the transmission LED lamp. The image is a maximum projection over a 172.36 µm z-stack of 63 images. Effective voxel size is 0.459 × 0.459 × 2.780 µm^3^. Scale bar, 100 µm. (**c**) PCA of GIP-Venus-positive (green) and negative (black) cell populations following bulk RNA-seq. (**d**, **e**) Differential *GIP* (**d**) and *Venus* (**e**) expression in Venus-positive (*n*=4) and negative (*n*=3) cell populations. Data are presented as mean ± SE; ***p<0.001 by two-tailed *t* test. (**f**) Representative FACS plot. GIP-Venus-positive and negative cells were isolated based on Venus fluorescence intensity, after selection of live DAPI-negative and DRAQ5-positive cells only. (**g**) Representative traces of perforated patch, whole-cell current clamp recording of a Venus-positive K cell held at −70 mV in response to the injection of short depolarising pulses (50 ms) of increasing amplitude in 7 pA increments, as indicated. (**h**) As shown in (**g**) using longer current injection pulses (500 ms) at 2 pA increments, as indicated. ex, exon; PAM, protospacer adjacent motif; PC, principal component

To generate GIP-Venus organoids, a P2A sequence followed by the *Venus* coding sequence was inserted to replace the stop codon of the *GIP* open reading frame in wild-type (WT) human duodenum-derived organoids by CRISPR-Cas9-mediated HDR (Fig. 1a). A similar strategy was used to generate a GIP-Epac-S-H187 reporter line. Sporadic yellow fluorescent cells with a pattern typical of EECs were observed in mature organoids (Fig. 1b). Dissociated GIP-Venus organoids were separated by FACS to isolate Venus-expressing K cells from non-fluorescent cells (Fig. 1f). Following RNA extraction and cDNA library preparation, bulk RNA-seq of Venus-positive K cells and Venus-negative cells was carried out and principal component analysis (PCA) revealed clear separation between the two populations (Fig. 1c). The RNA-seq dataset shows marked enrichment (~2000-fold) of both *GIP* and *Venus* genes in fluorescent compared with negative populations (Fig. 1d, e), confirming that Venus-positive cells are *GIP*-expressing K cells.

### Transcriptomic and peptidomic analysis of human K cells

**Fig. 2:**
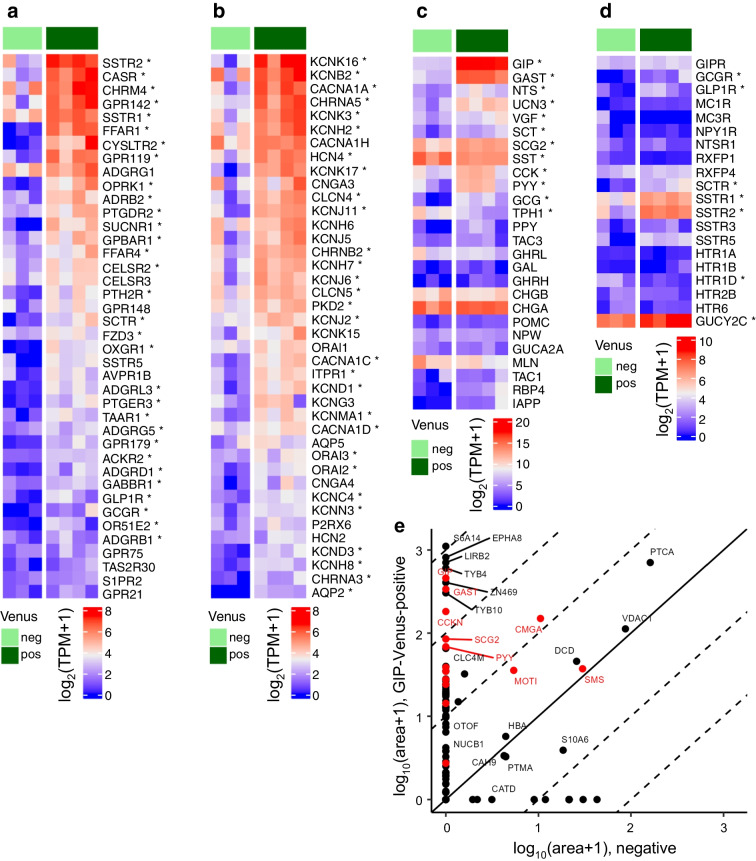
Transcriptomic and peptidomic characterisation of Venus-positive K cells. (**a**–**d**) Heatmaps showing: (**a**) top 40 highest expressed GPCRs; (**b**) top 40 ion channels and transporters; (**c**) gut peptides (plus tryptophane hydroxylase 1 [TPH1], the enzyme critical for serotonin production in enterochromaffin cells); (**d**) receptors for enteroendocrine hormones. *Significant differential expression between GIP-Venus K cells compared with the Venus-negative population (FDR<0.05). (**e**) LC-MS/MS peptidomic analysis of purified GIP-Venus positive and Venus-negative cells (the individual peptides detected are combined and associated to the parental protein, labelled by protein name [SwissProt] and expressed as mean peak area). FDR, false discovery rate; neg, negative; pos, positive; TPM, transcripts per million

Bulk RNA-seq revealed that Venus-positive K cells also exhibit enrichment of gastrin (*GAST*), cholecystokinin (*CCK*) and peptide YY (*PYY*) mRNA (Fig. 2c). Somatostatin (encoded by *SST*) was detected in K cells, but less than in the Venus-negative population (Fig. 2c). Ghrelin (*GHRL*) and secretin (*SCT*) were also detected at lower levels (Fig. 2c). Differential expression of GPCRs previously described to play a role in EEC nutrient sensing [17] was observed in human K cells (Fig. 2a). Amongst the GPCRs that are significantly differentially enriched, we identified *CASR* and *GPR142*, involved in AA sensing; *FFAR1/4* and *GPR119*, implicated in long-chain fatty acid (LCFA) and monoacylglycerol sensing, respectively; and *GPBAR1*, which mediates bile acid sensing. Other GPCRs involved in neurohormonal signalling and associated with obesity and insulin resistance, such as *OPRK1* [18] and *ADRB2* [19], and receptors for peptide hormones, including somatostatin (*SSTR1/2/5*) and secretin (*SCTR*) (Fig. 2a, d), were also enriched in K cells.

Mirroring previous observations in human GLP-1-secreting L cells [10], the GIP-Venus RNA-seq dataset showed differential expression of voltage-gated calcium channels important for action potential generation and vesicular exocytosis, including *CACNA1A* (P/Q type, Ca_v_2.1) and *CACNA1C* (L-type) (Fig. 2b) [20]. *CACNA1H* (T-type) was also highly expressed in GIP-Venus K cells, but not enriched. In contrast to L cells [10], the voltage-gated Na^+^ channel *SCN3A* (Na_v_1.3) was not enriched in Venus-positive K cells, although detected. Expression of the voltage-gated K^+^ channel *KCNB2* and hyperpolarisation-activated cyclic nucleotide-gated channel *HCN4* was enriched (Fig. 2b). LC-MS/MS peptidomic analysis of isolated Venus-positive K cells confirmed production of several gut hormone and neuroendocrine secretory peptides besides GIP, including gastrin, cholecystokinin (CCK), peptide YY (PYY), motilin, somatostatin (SST), chromogranin-A and secretogranin II (Fig. 2e).

### K cells are electrically active and secrete GIP upon glucose and α-MDG stimulation

**Fig. 3:**
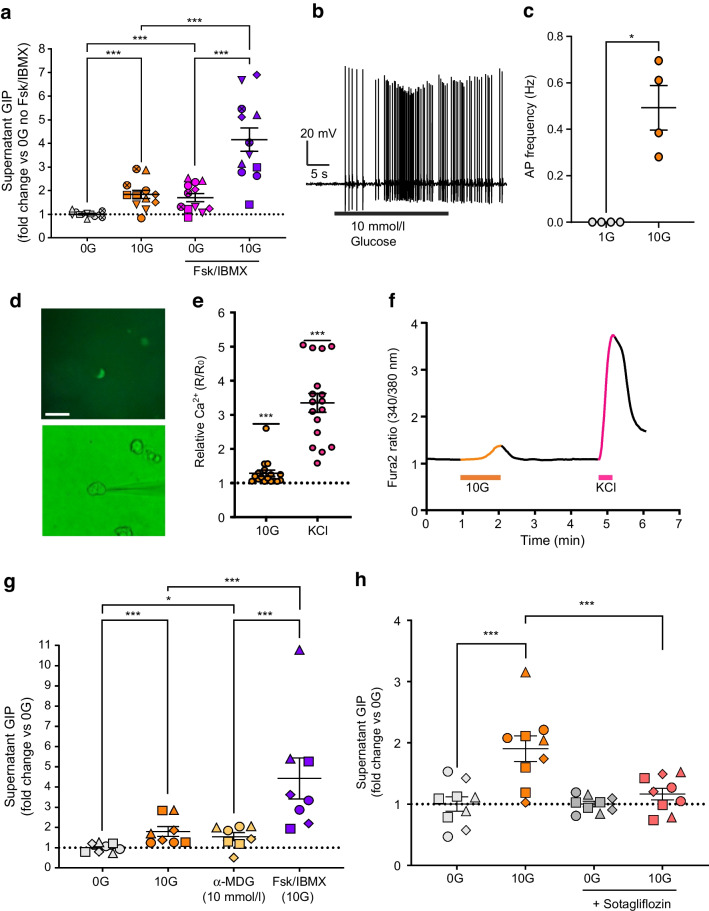
Glucose triggers firing of action potentials and GIP secretion in human K cells. (**a**) Secretion of GIP from GIP-Venus human duodenal organoids following incubation with glucose (10 mmol/l; 10G), in the presence or absence of Fsk (10 μmol/l) and IBMX (100 μmol/l), expressed as fold change vs basal condition (0 mmol/l glucose; 0G) measured in parallel (*n*=12 wells from six independent experiments; matching symbols indicate results from the same experiment). (**b**) Representative trace of perforated patch, whole-cell current clamp recording of a Venus-positive K cell initially perfused with 1 mmol/l glucose and exhibiting action potentials after perfusion with 10 mmol/l glucose, without current injection. (**c**) Mean action potential frequencies (Hz) of Venus-positive K cells recorded in 1 and 10 mmol/l glucose (G). (**d**) Images of Venus-positive K cells studied by perforated patch-clamp electrophysiology. K cells were identified by the expression of Venus (top panel) and patched using phase contrast (bottom panel). Scale bar, 50 μm. (**e**) Increase in intracellular calcium levels across different cells, shown as ratio between R (Fura-2 ratio during perfusion of stimulus) and R_0_ (Fura-2 ratio during perfusion of basal solution) (*n*=17 cells from nine independent experiments). (**f**) Representative Fura-2 (340/380 nm) ratio trace of a single K cell perfused with glucose (10 mmol/l, orange) and KCl (positive control; 70 mmol/l, pink). (**g**) Secretion of GIP from duodenal organoids in response to glucose (10 mmol/l) and α-MDG (10 mmol/l). Control solution (0G) contained 0 mmol/l glucose. Fsk (10 μmol/l) and IBMX (100 μmol/l) with 10 mmol/l glucose were used as positive control (*n*=8 wells from four independent experiments; matching symbols indicate results from the same experiment). (**h**) Inhibition of GIP release at 10 mmol/l glucose (10G) following 30 min pre- and 2 h co-incubation of duodenal organoids with sotagliflozin (5 μmol/l), expressed as fold change vs basal condition (0 mmol/l glucose; 0G) measured in parallel (*n*=9 wells from four independent experiments; matching symbols indicate results from the same experiment). Data are presented as mean ± SE. *p<0.05, ***p<0.001. (**a**, **g**, **h**) Linear regression and cluster-robust SE estimation with Huber–White SEs; (**c**) paired *t* test; (**e**) one-sample Wilcoxon test. AP, action potential; Fsk, forskolin

The K cells studied did not exhibit spontaneous action potential firing at low glucose (1 mmol/l), but action potentials were elicited in response to current injection (Fig. 1g, h), demonstrating that K cells are electrically excitable. The mean resting membrane potential was −50.8 ± 5.7 mV (*n*=8), action potential threshold was −25.7 ± 2.5 mV (*n*=4), action potential overshoot was +34.3 ± 5.9 mV (*n*=4) and action potential half width was 14.5 ± 2.1 ms (*n*=4). Treatment with 10 mmol/l glucose triggered spontaneous action potential firing in K cells (Fig. 3b), with significantly higher mean action potential frequency at 10 mmol/l glucose compared with baseline (1 mmol/l glucose) (from 0.0 ± 0.0 Hz to 0.5 ± 0.1 Hz, *n*=4) (Fig. 3c).

We further explored effects of glucose on GIP secretion in vitro and the potential underlying molecular mechanisms. Incubation of GIP-Venus organoids with glucose (10 mmol/l) triggered a 1.8 ± 0.2-fold (*n*=12) increase in GIP secretion, which was doubled in the presence of cAMP-raising agents forskolin (10 μmol/l) and IBMX (100 μmol/l) (Fig. 3a). Intracellular Ca^2+^ elevations were observed in 17/17 K cells perfused with glucose (10 mmol/l) (Fig. 3e, f). GIP secretion was also elicited by the SGLT1 substrate methyl α-d-glucopyranoside (α-MDG) (10 mmol/l; 1.5 ± 0.2-fold increase, *n*=8) (Fig. 3g). In the presence of the SGLT1/sodium glucose co-transporter 2 (SGLT2) inhibitor sotagliflozin, glucose-elicited GIP release was reduced from 1.9 ± 1.2-fold to 1.2 ± 0.1-fold (*p*<0.001; *n*=9) (Fig. 3h), supporting the idea that glucose-mediated GIP release is SGLT1-dependent.

### Stimulation of GIP secretion by GPCR agonists

**Fig. 4:**
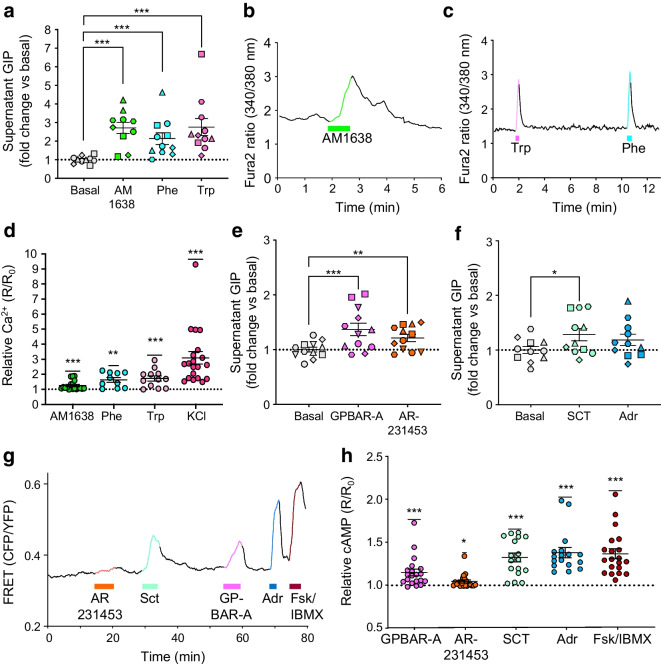
Stimulation of GIP release by AAs, LCFAs, bile acids and other small molecules. (**a**, **e**, **f**) Secretion of GIP from GIP-Venus human duodenal organoids in response to the stimuli indicated, expressed as fold change vs basal condition (1 mmol/l glucose) measured in parallel. The stimuli included AM1638 (10 μmol/l), phenylalanine (20 mmol/l), tryptophan (20 mmol/l), GPBAR-A (3 μmol/l), AR231453 (100 nmol/l), SCT (100 nmol/l) and adrenaline (30 μmol/l). All test solutions contained 1 mmol/l glucose (*n*=10–12 wells from 5–6 independent experiments; matching symbols indicate results from the same experiment). (**b**, **c**) Representative Fura-2 (340/380) ratio traces of single K cells perfused with AM1638 (10 μmol/l) and aromatic AAs phenylalanine and tryptophan (20 mmol/l), as indicated by the horizontal bars. (**d**) Mean data collected as in (**b**, **c**), shown as ratio between R (Fura-2 ratio during perfusion of stimulus) and R_0_ (Fura-2 ratio during perfusion of basal solution) (*n*=10–19 cells from 3–6 independent experiments). (**g**) Representative FRET (CFP/YFP) ratio trace of single K cell perfused with GPBAR-A (3 μmol/l), AR231453 (100 nmol/l), SCT (100 nmol/l), adrenaline (30 μmol/l) and positive control forskolin (Fsk)/IBMX (10 μmol/l/100 μmol/l), as indicated by the horizontal bars. (**h**) Mean data collected as in (**g**), shown as ratio between maximal CFP/YFP ratio (R) during perfusion of stimulus and maximal CFP/YFP ratio (R_0_) during perfusion of basal solution (*n*=17–19 from 4–5 independent experiments). Data are presented as mean ± SE. *p<0.05, **p<0.01, ***p<0.001. (**a**, **e**, **f**) Linear regression and cluster-robust SE estimation with Huber–White SEs; (**d**, **h**) one-sample Wilcoxon test. Adr, adrenaline

Secretion experiments were performed to explore functional expression of the nutrient- and metabolite-sensing receptors FFAR1, calcium-sensing receptor (CASR), G protein-coupled receptors 142 and 119 (GPR142, GPR119) and G protein-coupled bile acid receptor 1 (GPBAR1). We used a synthetic FFAR1 ligand, AM1638 (10 μmol/l), rather than an LCFA to avoid cross-reactivity with free fatty acid receptor 4 (FFAR4). AM1638 stimulated a significant increase in GIP secretion (2.7 ± 0.3-fold, *n*=10); the aromatic AAs phenylalanine and tryptophan (20 mmol/l) elicited 2.1 ± 0.3- (*n*=11) and 2.8 ± 0.5-fold (*n*=11) increases, respectively (Fig. 4a). These stimuli also promoted intracellular Ca^2+^ responses in K cells (Fig. 4b–d), presumably reflecting the G_q_-coupling of FFAR1, GPR142 and CASR. AM1638 elicited significant responses in 13/19 K cells, while phenylalanine and tryptophan triggered responses in 9/10 and 10/12 K cells, respectively (Fig. 4d).

Compared with AM1638 and the aromatic AAs, the GPBAR1 agonist GPBAR-A (3 μmol/l) and GPR119 agonist AR231453 (100 nmol/l) appeared to have less marked effects on GIP secretion, inducing 1.3 ± 0.1-fold (*n*=12) and 1.2 ± 0.1-fold (*n*=12) increases, respectively (Fig. 4e). Secretin (100 nmol/l) and adrenaline (epinephrine) (30 μmol/l) also had small effects, inducing only 1.3 ± 0.1-fold (*n*=12) and 1.2 ± 0.1-fold (*n*=12) increases, respectively (Fig. 4f). As GPBAR1, GPR119, secretin receptor (SCTR) and beta-2 adrenergic receptor (ADRB2) are G_s_-coupled, intracellular cAMP levels in K cells were monitored using the GIP-Epac-S-H187 line. Consistent with the detection of mRNA for these receptors, most tested G_s_-coupled stimuli induced marked increases in cAMP levels of GIP-Epac-S-H187 cells (Fig. 4g, h), with the exception of the GPR119 agonist AR231453, which evoked only modest responses. GPBAR-A provoked a significant increase in the CFP/YFP ratio, a measure of intracellular cAMP, in 12/19 GIP-Epac-S-H187 cells, whereas responses to AR231453 were observed in 6/19 cells. Secretin and adrenaline induced CFP/YFP responses in 15/17 and 17/17 GIP-Epac-S-H187 cells, respectively (Fig. 4h).

### Unravelling the role of GPR142 and CASR in AA sensing in K cells

**Fig. 5:**
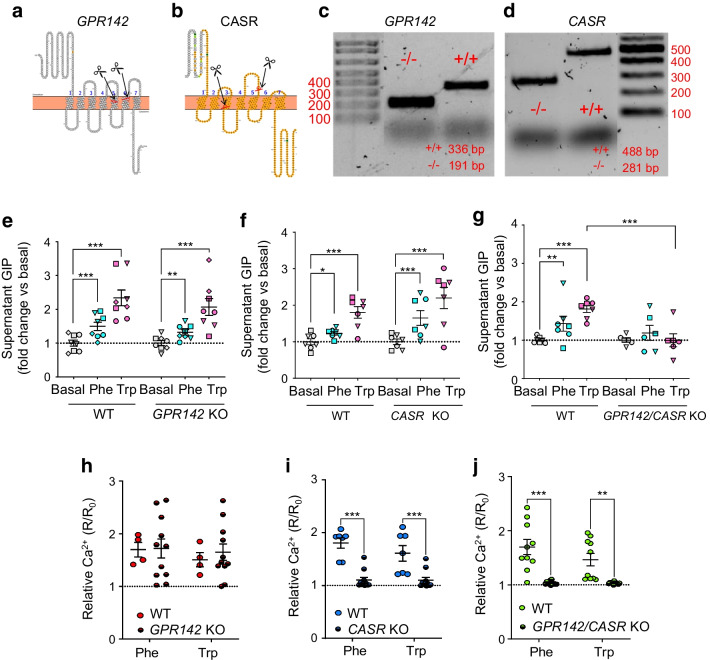
Unravelling the role of CASR and GPR142 in AA sensing in K cells. (**a**, **b**) Schematics representing *GPR142* and *CASR* CRISPR-Cas9 KO strategy. Sequences between the scissors represent deleted regions. The topological structures of the two receptors were generated using Protter (version 1.0; https://wlab.ethz.ch/protter/start/). (**c**, **d**) Representative agarose gels showing PCR genotyping results for WT (+/+) and homozygous (−/−) *GPR142* (**c**) and *CASR* (**d**) KO human GIP-Venus duodenal organoids; expected band sizes for WT and KO alleles are indicated in base pairs. (**e**–**g**) Secretion of GIP following stimulation with phenylalanine (20 mmol/l) and tryptophan (20 mmol/l) in WT and *GPR142* KO (**e**), *CASR* KO (**f**) and double KO (**g**) organoids, respectively. GIP release is expressed as fold change vs basal condition. All secretion experiments for KO lines were carried out in parallel with the WT line (*n*=6–8 wells from 3–4 independent experiments; matching symbols indicate results from the same experiment). (**h**–**j**) Increase in intracellular calcium levels in response to phenylalanine (20 mmol/l) and tryptophan (20 mmol/l) across K cells derived from WT and *GPR142* KO (**h**), *CASR* KO (**i**) and double KO (**j**) organoids, respectively. The increase is shown as ratio between R (Fura-2 ratio during perfusion of stimulus) and R_0_ (Fura-2 ratio during perfusion of basal solution) (*n*=4–11 cells from 3–4 independent experiments). Data are presented as mean ± SE. *p<0.05, **p<0.01, ***p<0.001. (**e**–**g**) Linear regression and cluster-robust SE estimation with Huber-White SEs; (**h**–**j**) two-way ANOVA with Sidak’s multiple comparisons

Two pairs of sgRNAs were used to delete transmembrane domains 5 and 6 and intracellular loop 3 of GPR142 (Fig. 5a) and intracellular and extracellular loops 2 and transmembrane domain 4 of CASR (Fig. 5b). Successful biallelic KO of the receptors was confirmed by PCR screening of surviving GIP-Venus organoids post gene editing (Fig. 5c, d).

*GPR142* KO GIP-Venus K cells did not show any significant impairment of intracellular Ca^2+^ responses to phenylalanine and tryptophan compared with WT K cells (Fig. 5h). Both phenylalanine and tryptophan also stimulated GIP secretion in *GPR142* KO organoids (1.3 ± 0.1- and 2.1 ± 0.2-fold [*n*=8 each], respectively), with no significant difference compared with WT organoids (1.5 ± 0.1- and 2.3 ± 0.2-fold [*n*=8 each], respectively) (Fig. 5e).

By contrast, intracellular Ca^2+^ responses to phenylalanine and tryptophan were significantly impaired in *CASR* KO K cells (Fig. 5i). Surprisingly, secretion of GIP from *CASR* KO organoids did not differ significantly from that of WT organoids, with both AAs significantly stimulating GIP secretion in the *CASR* KO (1.6 ± 0.2-fold increase by phenylalanine and 2.2 ± 0.3-fold increase by tryptophan [*n*=7 each]) (Fig. 5f).

Finally, we explored the effects of double *GPR142* and *CASR* KO on intracellular Ca^2+^ and GIP secretion. Ca^2+^ responses to phenylalanine and tryptophan were significantly impaired in double KO K cells (Fig. 5j). Double KO organoids also showed impaired GIP release in response to phenylalanine and tryptophan (1.1 ± 0.2- and 1.0 ± 0.2-fold increase, respectively [*n*=6–7]) (Fig. 5g).

## Discussion

The molecular characterisation of secretory mechanisms in K cells has relied heavily on mouse models and primary rodent intestinal cultures, in the absence of a human in vitro model. Using CRISPR-Cas9, we were able to insert the genes of fluorescent reporters at the 3′ end of the *GIP* gene in human duodenal organoids, enabling identification of living K cells in culture for downstream applications including FACS, RNA-seq, peptidomics, electrophysiology and live-cell imaging of Ca^2+^ and cAMP. Generation of the GIP-Venus and GIP-Epac-S-H187 reporter lines provides a breakthrough for characterising the cellular and molecular properties of human K cells in vitro.

Transcriptomic and peptidomic profiling of human K cells revealed expression and translation of an array of peptide hormones besides GIP. This includes gastrin and cholecystokinin, which aligns with previous observations made in neurogenin-3 (NEUROG3)-overexpressing human organoids [21]. Bulk RNA-seq revealed enriched expression of various nutrient- and hormone-sensing GPCRs and transporters in human K cells, aligning with previous observations showing mRNA enrichment of similar GPCRs and transporters in murine K cells by RT-PCR [22]. We report here that human K cells respond to a range of nutrients, hormones and small molecules, resulting in elevation of intracellular second messengers (Ca^2+^ and cAMP) and secretion of GIP. Importantly, we demonstrate for the first time that human K cells are electrically excitable and explore the role of AA-sensing receptors GPR142 and CASR in GIP secretion using CRISPR-Cas9 gene editing.

GIP secretion was stimulated by glucose and the non-metabolisable SGLT1 substrate α-MDG, and glucose-triggered GIP release was inhibited by sotagliflozin, an SGLT1/2 blocker used for management of diabetes and chronic kidney disease [23]. These results suggest that SGLT1 acts as the primary glucose sensor in human K cells, mirroring previous results from human physiological studies and mouse models. In humans, GIP secretion is triggered by oral but not intravenous administration of glucose [24, 25], and could also be stimulated by ingestion of 3-O-methyl glucose, a non-metabolisable SGLT1 substrate, but not by slowly absorbable saccharides [26], pointing towards the importance of sugar absorption. In mouse models, a number of pieces of evidence suggested that glucose-induced GIP secretion is due directly to the action of SGLT1: oral administration of α-MDG elevated circulating GIP levels [27]; in mouse primary intestinal cultures, the SGLT1 inhibitor phloridzin abolished glucose-triggered GIP release [22]; and in *Sglt1* KO mice the expected increase in circulating GIP following an oral glucose load was lost [28]. The mechanism of SGLT1-dependent glucose sensing is believed to involve the coupled entry of two Na^+^ ions with each glucose molecule, leading to membrane depolarisation as described previously in L cells [29, 30]. Consistent with this idea, action potential firing in human K cells was triggered by glucose elevation. Interestingly, we observed that the four fluorescent K cells recorded in this study did not exhibit spontaneous action potentials in 1 mmol/l glucose, unlike other EEC types from which we have recorded [10, 11]. With the low number of cells recorded, however, it is not possible to conclude whether this is a feature of human K cells, or a consequence of the specific culture conditions.

We studied the function of a variety of G_s_-coupled receptors identified in the RNA-seq analysis, including GPR119, GPBAR1, ADRB2 and SCTR. GIP secretion was modestly stimulated by the GPR119 agonist AR231453, with a corresponding increase in intracellular levels of cAMP in human K cells. This supports the finding that, in human volunteers, ingestion of C4-dietary oil, a prodrug for the GPR119 agonist 2-oleoyl glycerol, enhanced the GIP response to a carrot meal [31]. Similarly, oral gavage of WT mice with AR231453 stimulated GIP release which was impaired in *Gpr119* KO mice [32]. The GPBAR1 agonist GPBAR-A also increased K cell cAMP levels and GIP secretion from human organoids. The role of bile acids in the physiological modulation of GIP release remains unclear: while intraluminal administration of bile acids triggered GIP secretion in the perfused rat intestinal model [33], healthy volunteers taking the GPBAR1 agonist chenodeoxycholic acid surprisingly exhibited reduced meal-stimulated GIP release [34]. Adrenaline and secretin (SCT) had limited effects on GIP secretion in vitro, but robustly raised cAMP in the majority of the K cells assayed. β-Adrenergic stimulation has been shown previously to increase plasma GIP levels in healthy volunteers [35], but effects of SCT have been tested only in the context of a meal, when they were found not to affect postprandial GIP levels in healthy individuals [36]. Organoid GIP secretory responses to G_s_-coupled receptor activation were noticeably smaller than those to G_q_-coupled receptor agonism, but the secretion experiments were performed in 1 mmol/l glucose where spontaneous electrical activity was low. As cAMP sensitises exocytotic machinery to raised Ca^2+^ in other endocrine cell types [37], the results suggest that K cell Ca^2+^ levels under these conditions were close to the threshold for cAMP-dependent secretion. While we did not test the effect of predominantly G_i_-coupled receptors in this study, including the enriched SST receptor subtypes and the dynorphin (κ-opioid) receptor encoded by *OPRK1*, we would expect these to inhibit stimulated GIP secretion, in line with previous observations on mouse K cells, which are inhibited by SST and endocannabinoid 1 receptor (CB1) agonist [38]. Given that Venus-positive K cells also co-express *SST*, this could act as an autocrine break on GIP secretion, although we cannot exclude a contribution of *SST*-expressing D cells, which would underlie the *SST* signal in the Venus-negative population. The predominantly G_s_-coupled *GIPR* itself was detectable, opening the possibility that there is also an autocrine feed-forward loop in operation in K cells. However, previous research has demonstrated the importance of SST for GIP secretion, as SST-neutralising antibodies increased glucose-stimulated GIP secretion in both the STC-1 cell line model [39] and elutriated canine K cells [40].

G_q_ activation triggered robust elevation of Ca^2+^ and GIP secretion in human K cells, including agonists for FFAR1, CASR and GPR142. AM1638 was a strong stimulus of Ca^2+^ elevation and GIP secretion, suggesting a role for FFAR1 in LCFA-mediated GIP release in humans. This coincides with previous reports that plasma GIP is elevated following oral olive oil or corn oil gavage in WT mice, which was blunted in *Ffar1* KO animals [41]. FFAR4 agonism was not tested because K cell expression of *FFAR4* was fourfold lower than *FFAR1*, and because we previously observed no responses to the FFAR4-specific agonists TUG891 (1 μmol/l) or compound A (1 μmol/l) in human *MLN*-labelled duodenal organoid cells which expressed >3 times more *FFAR4* than the K cells in the current study [11]. Phenylalanine and tryptophan, known ligands for CASR and GPR142, similarly triggered robust elevation of K cell Ca^2+^ and GIP secretion from human organoids, supporting reports that intraduodenal delivery of an AA mix [42] or glutamine [43] stimulated GIP secretion in healthy volunteers. We further investigated the molecular mechanism underlying AA-triggered GIP release in human organoids, as both GPR142 and CASR have been proposed as K cell AA sensors. In mice, tryptophan-triggered GIP secretion was abolished in *Gpr142* KO animals [44], and in perfused pig duodenum studies, phenylalanine-triggered GIP secretion was blunted by the CASR antagonist NPS-2143 [45]. We therefore examined the effects of knocking out *CASR* or *GPR142* alone or in combination in GIP-Venus organoids. Unlike in mouse studies, *GPR142* KO in human organoids had no effect on either GIP secretion or K cell Ca^2+^ responses to tryptophan or phenylalanine, suggesting that GPR142 is not essential for AA-mediated GIP stimulation in humans. *CASR* KO reduced K cell Ca^2+^ responses to tryptophan and phenylalanine but did not impair AA-triggered GIP secretion, so while CASR might play a role in acute Ca^2+^ responses to these AAs, other mechanisms seem to contribute to AA-stimulated GIP release over 2 h. In double KO organoids acute Ca^2+^-responses to tryptophan and phenylalanine were further diminished, and tryptophan-induced GIP release was significantly reduced. Our results therefore suggest that both CASR and GPR142 are needed in K cells to mount a full response to aromatic AAs, with CASR playing a key role in mediating the Ca^2+^ responses. Even though secretory responses to phenylalanine and tryptophan did not reach significance in the double KO, apparent responses in some experiments suggest the presence of additional pathways, potentially involving AA absorption and metabolism, and further pharmacological and KO studies are necessary to elucidate the potential role of AA transporters in GIP secretion from human K cells. Responses to other AAs such as branched chain AAs were not tested in the current study, as previous results in humans with leucine and isoleucine were inconsistent [46–48].

### Conclusions

Human K cells are directly responsive to a range of nutritional-related stimuli, including glucose, fatty acids and aromatic AAs. Differential expression of a number of nutrient-sensing GPCRs and transporters was identified in GIP-Venus cells, shedding light on potential molecular mechanisms orchestrating nutrient and small molecule sensing in human K cells. GIP secretion was strongly elicited by SGLT1 substrates, FFAR1 agonism and aromatic AAs. Glucose-mediated GIP release was mediated by SGLT1, as demonstrated by its sensitivity to the SGLT1/2 inhibitor sotagliflozin, correlating with the glucose-dependent membrane depolarisation and action potential firing evident in human K cell electrophysiological recordings. The application of CRISPR-Cas9-mediated gene KO in human intestinal organoid models, used here to demonstrate the joint contribution of GPR142 and CASR to AA-triggered GIP release, promises to be a powerful tool to dissect the physiological importance of receptors, ion channels and transporters for which specific pharmacological tools are lacking. Characterising the mechanisms underlying K cell nutrient sensing will facilitate our understanding of the human gut–brain–pancreatic axis and how it could be targeted for the treatment of metabolic diseases.

## Supplementary Information

Below is the link to the electronic supplementary material.

ESM (PDF 157 KB)

## Data Availability

RNA-seq data are deposited in the National Center for Biotechnology Information–Gene Expression Omnibus (NCBI GEO) repository (GSE271017). Mass spectrometry proteomics data are deposited to the ProteomeXchange Consortium via the PRIDE partner repository (PXD052659).

## Abbreviations

AA: Amino acid
ADRB2: β-2 Adrenergic receptor
CASR: Calcium-sensing receptor
CFP: Cyan fluorescent protein
EEC: Enteroendocrine cell
FFAR1: Free fatty acid receptor 1
FFAR4: Free fatty acid receptor 4
FRET: Fluorescence resonance energy transfer
GIP: Glucose-dependent insulinotropic polypeptide
GIPR: Glucose-dependent insulinotropic polypeptide receptor
GLP-1: Glucagon-like peptide 1
GLP1R: Glucagon-like peptide 1 receptor
GPBAR1: G protein-coupled bile acid receptor 1
GPCR: G protein-coupled receptor
GPR119: G protein-coupled receptor 119
GPR142: G protein-coupled receptor 142
HDR: Homology-directed repair
IBMX: 3-isobutyl-1-methylxanthine
KO: Knockout
LCFA: Long-chain fatty acid
α-MDG: Methyl α-d-glucopyranoside
P2A: Picornaviral peptide-2A sequence
PCA: Principal component analysis
SCT: Secretin
SCTR: Secretin receptor
SGLT1: Sodium glucose co-transporter 1
SGLT2: Sodium glucose co-transporter 2
sgRNA: Single guide RNA
SST: Somatostatin
WT: Wild-type
YFP: Yellow fluorescent protein

## References

[1] Guccio N, Gribble FM, Reimann F (2022) Glucose-dependent insulinotropic polypeptide-a postprandial hormone with unharnessed metabolic potential. Annu Rev Nutr 42:21–44. 10.1146/annurev-nutr-062320-113625 35609956 10.1146/annurev-nutr-062320-113625

[2] O’Neil PM, Birkenfeld AL, McGowan B et al (2018) Efficacy and safety of semaglutide compared with liraglutide and placebo for weight loss in patients with obesity: a randomised, double-blind, placebo and active controlled, dose-ranging, phase 2 trial. Lancet 392(10148):637–649. 10.1016/S0140-6736(18)31773-2 30122305 10.1016/S0140-6736(18)31773-2

[3] Rubino D, Abrahamsson N, Davies M et al (2021) Effect of continued weekly subcutaneous Semaglutide vs Placebo on weight loss maintenance in adults with overweight or obesity: The STEP 4 Randomized Clinical Trial. JAMA 325(14):1414–1425. 10.1001/jama.2021.3224 33755728 10.1001/jama.2021.3224 PMC7988425

[4] Nauck MA, Heimesaat MM, Orskov C, Holst JJ, Ebert R, Creutzfeldt W (1993) Preserved incretin activity of glucagon-like peptide 1 [7-36 amide] but not of synthetic human gastric inhibitory polypeptide in patients with type-2 diabetes mellitus. J Clin Invest 91(1):301–307. 10.1172/JCI116186 8423228 10.1172/JCI116186 PMC330027

[5] Jastreboff AM, Aronne LJ, Ahmad NN et al (2022) Tirzepatide once weekly for the treatment of obesity. N Engl J Med 387(3):205–216. 10.1056/NEJMoa2206038 35658024 10.1056/NEJMoa2206038

[6] Nauck M, Stöckmann F, Ebert R, Creutzfeldt W (1986) Reduced incretin effect in type 2 (non-insulin-dependent) diabetes. Diabetologia 29(1):46–52. 10.1007/BF02427280 3514343 10.1007/BF02427280

[7] Elliott RM, Morgan LM, Tredger JA, Deacon S, Wright J, Marks V (1993) Glucagon-like peptide-1 (7–36)amide and glucose-dependent insulinotropic polypeptide secretion in response to nutrient ingestion in man: acute post-prandial and 24-h secretion patterns. J Endocrinol 138(1):159–166. 10.1677/joe.0.1380159 7852887 10.1677/joe.0.1380159

[8] Calbet JA, Holst JJ (2004) Gastric emptying, gastric secretion and enterogastrone response after administration of milk proteins or their peptide hydrolysates in humans. Eur J Nutr 43(3):127–139. 10.1007/s00394-004-0448-4 15168035 10.1007/s00394-004-0448-4

[9] Sato T, Vries RG, Snippert HJ et al (2009) Single Lgr5 stem cells build crypt-villus structures in vitro without a mesenchymal niche. Nature 459(7244):262–265. 10.1038/nature07935 19329995 10.1038/nature07935

[10] Goldspink DA, Lu VB, Miedzybrodzka EL et al (2020) Labeling and characterization of human GLP-1-Secreting L-cells in primary ileal organoid culture. Cell Rep 31(13):107833. 10.1016/j.celrep.2020.107833 32610134 10.1016/j.celrep.2020.107833 PMC7342002

[11] Miedzybrodzka EL, Foreman RE, Lu VB et al (2021) Stimulation of motilin secretion by bile, free fatty acids, and acidification in human duodenal organoids. Mol Metab 54:101356. 10.1016/j.molmet.2021.101356 34662713 10.1016/j.molmet.2021.101356 PMC8590067

[12] Fujii M, Matano M, Toshimitsu K et al (2018) Human intestinal organoids maintain self-renewal capacity and cellular diversity in niche-inspired culture condition. Cell Stem Cell 23(6):787-793 e786. 10.1016/j.stem.2018.11.016 30526881 10.1016/j.stem.2018.11.016

[13] Klarenbeek J, Goedhart J, van Batenburg A, Groenewald D, Jalink K (2015) Fourth-generation epac-based FRET sensors for cAMP feature exceptional brightness, photostability and dynamic range: characterization of dedicated sensors for FLIM, for ratiometry and with high affinity. PLoS One 10(4):e0122513. 10.1371/journal.pone.0122513 25875503 10.1371/journal.pone.0122513 PMC4397040

[14] Love MI, Huber W, Anders S (2014) Moderated estimation of fold change and dispersion for RNA-seq data with DESeq2. Genome Biol 15(12):550. 10.1186/s13059-014-0550-8 25516281 10.1186/s13059-014-0550-8 PMC4302049

[15] Brighton CA, Rievaj J, Kuhre RE et al (2015) Bile acids trigger GLP-1 release predominantly by accessing basolaterally located g protein-coupled bile acid receptors. Endocrinology 156(11):3961–3970. 10.1210/en.2015-1321 26280129 10.1210/en.2015-1321 PMC4606749

[16] Friedlander RS, Moss CE, Mace J et al (2011) Role of phosphodiesterase and adenylate cyclase isozymes in murine colonic glucagon-like peptide 1 secreting cells. Br J Pharmacol 163(2):261–271. 10.1111/j.1476-5381.2010.01107.x 21054345 10.1111/j.1476-5381.2010.01107.x PMC3087130

[17] Gribble FM, Reimann F (2019) Function and mechanisms of enteroendocrine cells and gut hormones in metabolism. Nat Rev Endocrinol 15(4):226–237. 10.1038/s41574-019-0168-8 30760847 10.1038/s41574-019-0168-8

[18] Romero-Pico A, Novelle MG, Al-Massadi O et al (2022) Kappa-opioid receptor blockade ameliorates obesity caused by estrogen withdrawal via promotion of energy expenditure through mTOR pathway. Int J Mol Sci 23(6):3118. 10.3390/ijms23063118 35328539 10.3390/ijms23063118 PMC8953356

[19] Mitra SR, Tan PY, Amini F (2019) Association of ADRB2 rs1042713 with obesity and obesity-related phenotypes and its interaction with dietary fat in modulating glycaemic indices in Malaysian adults. J Nutr Metab 2019:8718795. 10.1155/2019/8718795 31007954 10.1155/2019/8718795 PMC6441509

[20] Gilon P, Chae HY, Rutter GA, Ravier MA (2014) Calcium signaling in pancreatic beta-cells in health and in Type 2 diabetes. Cell Calcium 56(5):340–361. 10.1016/j.ceca.2014.09.001 25239387 10.1016/j.ceca.2014.09.001

[21] Beumer J, Puschhof J, Bauza-Martinez J et al (2020) High-resolution mRNA and secretome atlas of human enteroendocrine cells. Cell 181(6):1291-1306 e1219. 10.1016/j.cell.2020.04.036 32407674 10.1016/j.cell.2020.04.036

[22] Parker HE, Habib AM, Rogers GJ, Gribble FM, Reimann F (2009) Nutrient-dependent secretion of glucose-dependent insulinotropic polypeptide from primary murine K cells. Diabetologia 52(2):289–298. 10.1007/s00125-008-1202-x 19082577 10.1007/s00125-008-1202-x PMC4308617

[23] Bhatt DL, Szarek M, Pitt B et al (2021) Sotagliflozin in patients with diabetes and chronic kidney disease. N Engl J Med 384(2):129–139. 10.1056/NEJMoa2030186 33200891 10.1056/NEJMoa2030186

[24] Cataland S, Crockett SE, Brown JC, Mazzaferri EL (1974) Gastric inhibitory polypeptide (GIP) stimulation by oral glucose in man. J Clin Endocrinol Metab 39(2):223–228. 10.1210/jcem-39-2-223 4423791 10.1210/jcem-39-2-223

[25] Dupre J, Ross SA, Watson D, Brown JC (1973) Stimulation of insulin secretion by gastric inhibitory polypeptide in man. J Clin Endocrinol Metab 37(5):826–828. 10.1210/jcem-37-5-826 4749457 10.1210/jcem-37-5-826

[26] Wu T, Zhao BR, Bound MJ et al (2012) Effects of different sweet preloads on incretin hormone secretion, gastric emptying, and postprandial glycemia in healthy humans. Am J Clin Nutr 95(1):78–83. 10.3945/ajcn.111.021543 22158727 10.3945/ajcn.111.021543

[27] Moriya R, Shirakura T, Ito J, Mashiko S, Seo T (2009) Activation of sodium-glucose cotransporter 1 ameliorates hyperglycemia by mediating incretin secretion in mice. Am J Physiol Endocrinol Metab 297(6):E1358-1365. 10.1152/ajpendo.00412.2009 19808907 10.1152/ajpendo.00412.2009

[28] Gorboulev V, Schurmann A, Vallon V et al (2012) Na(+)-D-glucose cotransporter SGLT1 is pivotal for intestinal glucose absorption and glucose-dependent incretin secretion. Diabetes 61(1):187–196. 10.2337/db11-1029 22124465 10.2337/db11-1029 PMC3237647

[29] Gribble FM, Williams L, Simpson AK, Reimann F (2003) A novel glucose-sensing mechanism contributing to glucagon-like peptide-1 secretion from the GLUTag cell line. Diabetes 52(5):1147–1154. 10.2337/diabetes.52.5.1147 12716745 10.2337/diabetes.52.5.1147

[30] Parker HE, Adriaenssens A, Rogers G et al (2012) Predominant role of active versus facilitative glucose transport for glucagon-like peptide-1 secretion. Diabetologia 55(9):2445–2455. 10.1007/s00125-012-2585-2 22638549 10.1007/s00125-012-2585-2 PMC3411305

[31] Mandoe MJ, Hansen KB, Windelov JA et al (2018) Comparing olive oil and C4-dietary oil, a prodrug for the GPR119 agonist, 2-oleoyl glycerol, less energy intake of the latter is needed to stimulate incretin hormone secretion in overweight subjects with type 2 diabetes. Nutr Diabetes 8(1):2. 10.1038/s41387-017-0011-z 29330461 10.1038/s41387-017-0011-z PMC6199285

[32] Chu ZL, Carroll C, Alfonso J et al (2008) A role for intestinal endocrine cell-expressed g protein-coupled receptor 119 in glycemic control by enhancing glucagon-like Peptide-1 and glucose-dependent insulinotropic Peptide release. Endocrinology 149(5):2038–2047. 10.1210/en.2007-0966 18202141 10.1210/en.2007-0966

[33] Kuhre RE, WewerAlbrechtsen NJ, Larsen O et al (2018) Bile acids are important direct and indirect regulators of the secretion of appetite- and metabolism-regulating hormones from the gut and pancreas. Mol Metab 11:84–95. 10.1016/j.molmet.2018.03.007 29656109 10.1016/j.molmet.2018.03.007 PMC6001409

[34] McGlone ER, Malallah K, Cuenco J et al (2021) Differential effects of bile acids on the postprandial secretion of gut hormones: a randomized crossover study. Am J Physiol Endocrinol Metab 320(4):E671–E679. 10.1152/ajpendo.00580.2020 33459181 10.1152/ajpendo.00580.2020

[35] Flaten O, Sand T, Myren J (1982) Beta-adrenergic stimulation and blockade of the release of gastric inhibitory polypeptide and insulin in man. Scand J Gastroenterol 17(2):283–288. 10.3109/00365528209182054 6127792 10.3109/00365528209182054

[36] Brandler J, Miller LJ, Wang XJ et al (2020) Secretin effects on gastric functions, hormones and symptoms in functional dyspepsia and health: randomized crossover trial. Am J Physiol Gastrointest Liver Physiol 318(4):G635–G645. 10.1152/ajpgi.00371.2019 32036693 10.1152/ajpgi.00371.2019 PMC7191464

[37] Skelin M, Rupnik M (2011) cAMP increases the sensitivity of exocytosis to Ca^2^ + primarily through protein kinase A in mouse pancreatic beta cells. Cell Calcium 49(2):89–99. 10.1016/j.ceca.2010.12.005 21242000 10.1016/j.ceca.2010.12.005

[38] Moss CE, Marsh WJ, Parker HE et al (2012) Somatostatin receptor 5 and cannabinoid receptor 1 activation inhibit secretion of glucose-dependent insulinotropic polypeptide from intestinal K cells in rodents. Diabetologia 55(11):3094–3103. 10.1007/s00125-012-2663-5 22872212 10.1007/s00125-012-2663-5 PMC3464380

[39] Kieffer TJ, Huang Z, McIntosh CH, Buchan AM, Brown JC, Pederson RA (1995) Gastric inhibitory polypeptide release from a tumor-derived cell line. Am J Physiol 269(2 Pt 1):E316-322. 10.1152/ajpendo.1995.269.2.E316 7653549 10.1152/ajpendo.1995.269.2.E316

[40] Kieffer TJ, Buchan AM, Barker H, Brown JC, Pederson RA (1994) Release of gastric inhibitory polypeptide from cultured canine endocrine cells. Am J Physiol 267(4 Pt 1):E489-496. 10.1152/ajpendo.1994.267.4.E489 7943296 10.1152/ajpendo.1994.267.4.E489

[41] Ekberg JH, Hauge M, Kristensen LV et al (2016) GPR119, a major enteroendocrine sensor of dietary triglyceride metabolites coacting in synergy with FFA1 (GPR40). Endocrinology 157(12):4561–4569. 10.1210/en.2016-1334 27779915 10.1210/en.2016-1334 PMC7212052

[42] Thomas FB, Mazzaferri EL, Crockett SE, Mekhjian HS, Gruemer HD, Cataland S (1976) Stimulation of secretion of gastric inhibitory polypeptide and insulin by intraduodenal amino acid perfusion. Gastroenterology 70(4):523–527. 10.1016/S0016-5085(76)80489-1 815125

[43] Greenfield JR, Farooqi IS, Keogh JM et al (2009) Oral glutamine increases circulating glucagon-like peptide 1, glucagon, and insulin concentrations in lean, obese, and type 2 diabetic subjects. Am J Clin Nutr 89(1):106–113. 10.3945/ajcn.2008.26362 19056578 10.3945/ajcn.2008.26362 PMC4340573

[44] Rudenko O, Shang J, Munk A et al (2019) The aromatic amino acid sensor GPR142 controls metabolism through balanced regulation of pancreatic and gut hormones. Mol Metab 19:49–64. 10.1016/j.molmet.2018.10.012 30472415 10.1016/j.molmet.2018.10.012 PMC6323244

[45] Feng J, Kang C, Wang C, Ding L, Zhu W, Hang S (2019) L-phenylalanine increased gut hormone secretion through calcium-sensing receptor in the porcine duodenum. Animals (Basel) 9(8):476. 10.3390/ani9080476 31344840 10.3390/ani9080476 PMC6719913

[46] Cleator IG, Gourlay RH (1975) Release of immunoreactive gastric inhibitory polypeptide (IR-GIP) by oral ingestion of food substances. Am J Surg 130(2):128–135. 10.1016/0002-9610(75)90360-8 1098505 10.1016/0002-9610(75)90360-8

[47] Newmire DE, Rivas E, Deemer SE, Willoughby DS, Ben-Ezra V (2019) The impact of a large bolus dose of l-leucine and l-isoleucine on enteroendocrine and pancreatic hormones, and glycemia in healthy, inactive adults. Nutrients 11(11):2650. 10.3390/nu11112650 31689951 10.3390/nu11112650 PMC6893504

[48] Ullrich SS, Fitzgerald PC, Schober G, Steinert RE, Horowitz M, Feinle-Bisset C (2016) Intragastric administration of leucine or isoleucine lowers the blood glucose response to a mixed-nutrient drink by different mechanisms in healthy, lean volunteers. Am J Clin Nutr 104(5):1274–1284. 10.3945/ajcn.116.140640 27655440 10.3945/ajcn.116.140640

