# Supplementary material for: Molecular mechanisms underlying glucose-dependent insulinotropic polypeptide secretion in human duodenal organoids
Source: Diabetologia. 2024 Oct 23;68(1):217–30. doi: 10.1007/s00125-024-06293-3 (PMC11663192; doi:10.1007/s00125-024-06293-3)
Supplement: Supplementary file 1 — ESM (PDF 157 KB) [file 125_2024_6293_MOESM1_ESM.pdf]

**Supplementary Methods for "Molecular mechanisms underlying glucose-dependent insulinotropic polypeptide secretion in human duodenal organoids" by Guccio et al:**

***Fluorescence-activated cell sorting (FACS)***

FACS was performed as previously described [1]. In brief, differentiated GIP-Venus organoids were chemically digested and mechanically triturated to single cells. Hanks' Balanced Salt Solution (without $Ca^{2+}$ or $Mg^{2+}$, Sigma-Aldrich) supplemented with 10 μmol/l Rho-kinase (ROCK) inhibitor Y-27632 and either 10% fetal bovine serum (FBS) or 0.1% bovine serum albumin (for RNA and peptide extraction, respectively) was used to resuspend the cells. Cells were stained with DNA dyes 2 mg/ml DAPI (cell impermeant, preferentially labelling dead cells) and 5 mmol/l DRAQ5 (BD Biosciences, Wokingham, UK) (cell permeant, also labelling live cells) and sorted using a BD FACSMelody cell sorter (BD Biosciences). Both Venus-positive and Venus-negative populations were collected into either RLT+ buffer (Qiagen, Manchester, UK) supplemented with 1% β-mercaptoethanol (1.5-10 x $10^3$ cells collected per sort) for RNA extraction or into 6 mol/l guanidine hydrochloride (10 x $10^3$ cells collected per sort) for peptide extraction.

***RNA seq***

RNA extraction was executed using RNAeasy Micro Plus kit (Qiagen). RNA 6000 Pico Kit and Bioanalyser 2000 (Agilent, Stockport, UK) was used to quantify and for quality control of extracted RNA. Generation of cDNA libraries was carried out with 2 ng input RNA (RIN 7-9) using the SMARTer Stranded Total RNA-Seq v2 Pico Input Mammalian kit (Takara Bio, London, UK) with fourteen PCR amplification cycles. Libraries were pooled to 4 nmol/l final concentration and single-end 50 bases sequenced on a HiSeq 4000 (Illumina, Cambridge, UK). Quality and adaptor trimming of sequenced transcripts was performed using cutadapt

(version 3.4) [2]. Demultiplex (version 1.2.1) [3] was used to demultiplex transcripts using the i7 Illumina index located at the 5' end. STAR (version 2.7.3a) [4] was used to align transcripts to the human genome (GRCh38). Raw counts were generated using featureCounts (version 2.0.3) [5]. Quality control was performed using FastQC (version 0.11.9) [6]. Differential gene expression analysis was performed in RStudio using DESeq2 (version 1.42.0) [7]. Gene annotation was obtained from the Ensembl dataset held in BiomaRt (version 2.58.2) [8, 9], combined with gene ontology annotation from goseq (version 1.54.0) [10]. Receptor and ion channel lists were generated from the International Union of Basic and Clinical Pharmacology (IUPHAR) "targets and families" list (accessed on 7 Jan 2020).

***Peptidomic analysis***

Sorted cells in 6 mol/l guanidine hydrochloride underwent 3 freeze-thaw cycles. 50 µL of 5 ng/mL bovine insulin in 1% (v/v in water) FA (formic acid) was added to the lysates, mixed, and diluted to 1 mL total volume with 0.1% (v/v) FA. HLB PRiME µElution solid-phase extraction plate (Waters, Wilmslow, UK) was employed to extract samples; ultimate 3000 nano-LC system (Thermo Fisher Scientific, Hemel Hempstead, UK) coupled to a Q-Exactive Plus Orbitrap mass spectrometer (Thermo Fisher Scientific) was used for sample analysis after reduction and alkylation, as previously described [11].

40 µl of each sample was loaded onto a 0.3 × 5 mm peptide trap column (Thermo Fisher Scientific), using a flow rate of 30 µl/min, and washed for 15 min before switching in line with a 0.075 × 250 mm nano easy column (Thermo Fisher Scientific), with a flow rate of 300 nl/min. The temperature for both nano trap columns was set to 45°C. The mobile phases were A: 0.1% (v/v) FA in water and B: 0.1% FA (v/v) in 80:20 ACN:water. Initial conditions were 2.5% B and kept for 15 min. A 90-minute ramp was employed to transition to 50% B, and the column was then washed using 90% B over a period of 20 min before returning to starting conditions for a further 20 mins, with the entire run taking 130 min in total. A spray voltage of 1.8 kV and an S-lens setting of 70 V were used to perform positive nano electrospray analysis. Using a

resolution of 75,000, a full scan range of 400–1600 m/z was performed prior to selection of the top 10 ions of each spectrum for MS/MS analysis. Existing ions selected for fragmentation were included to an exclusion list for 30 s.

PEAKS (version 8.5, Bioinformatics Solutions Inc, Waterloo, Canada) was used to match LC-MS/MS files against the Human Uniprot database (accessed, June 2022) and Xcalibur (version 4.3.73.11, Thermo Fisher Scientific) was used to perform peak integration. Search parameters included a no-enzyme setting, precursor (10 ppm) and production (0.05 Da) tolerances, a fixed modification of carbamidomethylation on cysteine residues, and variable modifications of methionine oxidation, N-terminal pyroglutamate, N-terminal acetylation, and C-terminal amidation. 1% false discovery rate and at least one unique peptide were used as parameters to filter the data. Peak area intensity of parental proteins in sorted cells was calculated in PEAKS. Statistical analysis of peak areas was performed using Perseus (version 2.0.11.0, https://maxquant.net/perseus/). The number of peptide fragments were summed per associated peptide prior to downstream plot generation.

***Calcium imaging***

Live single-cell calcium imaging of Venus-expressing K-cells was performed as previously described [12-14]. Briefly, differentiated organoids were proteolytically dissociated into single cells or small cell clusters, seeded on 35 mm glass-bottom dishes (MatTek, Ashland, MA, USA) precoated with 2-4% Matrigel (Corning) and incubated overnight. At time of imaging, the cultures were washed with saline buffer, loaded with fura2-AM (5 µmol/l) (Thermo Fisher Scientific, Bishops Stortsford, UK) in the presence of 1 mmol/l glucose in saline buffer and mounted on a 40X oil-objective on a fluorescence inverted microscope (Olympus IX71, Southall, UK). Fura2 emission at 340 and 380 nm excitation was recorded every 2 s while the cells were continuously perfused with saline buffer, in the presence or the absence of test reagents, using Metafluor software (Molecular Devices, UK) and an Orca-ER CCD camera

(Hamamatsu, Welwyn Garden City, UK). The fura2 ratios (340/380 nm) were calculated following background subtraction, and the responses are presented as fold change between the maximum fura2 ratio value recorded during stimulus perfusion and the maximum (baseline) fura2 ratio value recorded during perfusion saline buffer 1 min prior the onset of stimulus.

**cAMP imaging**

cAMP-dependent FRET imaging was performed on differentiated GIP-Epac-S-H187 organoids as described previously [15]. Cells were prepared as for $Ca^{2+}$-imaging and mounted on a fluorescence inverted microscope (Olympus IX71, Southall, UK) as above. Readouts for intracellular cAMP levels were recorded every 5 s while the cells were continuously perfused with saline buffer, in the presence or absence of test reagents, with Metafluor software (Molecular Devices, UK). Cells were excited at 435 nm, emissions of YFP and CFP were recorded using an optosplit (470/24 nm and 535/30 nm emission filters, respectively; Cairn Research, Faversham, UK) before the Orca-ER camera (Hamamatsu, Welwyn Garden City, UK), and CFP/YFP ratios were calculated after background subtraction. Responses are presented as fold change between the maximum CFP/YFP ratio value recorded during perfusion with baseline saline buffer 1 min prior the onset of stimulus and the maximum CFP/YFP ratio value recorded during perfusion of stimulus.

***Electrophysiological recordings***

2D cell cultures of human GIP-Venus organoids were prepared as described above the day before patch clamp recordings of individual Venus+ cells were performed at room temperature (20-24 $^{o}$C). Bath solution (saline buffer) was perfused at a constant rate using a custom-made gravity fed perfusion system. Perforated patch clamp recordings were performed using either an Axopatch 200B amplifier (Molecular Devices, UK) connected through a Digidata 1440A A/D converter (Molecular Devices), using pclamp software (version 10.7.03, Molecular Devices) or a HEKA (Reutlingen, Germany) EPC10 using patchmaster software (version 2x90.1,

HEKA). Microelectrodes were pulled from borosilicate glass (1.5 mm OD, 1.17 mm ID; Harvard apparatus, Edenbridge, UK) using a PC-100 Narishige microelectrode puller (Narishige, Japan) and the tips were coated with refined yellow beeswax. Electrodes were fire polished using an MF-830 microforge (Narishige, Japan) with a resistance of 2-3 MΩ when filled with internal pipette solution. A silver/AgCl ground wire connected to the bath solution via a 3 mol/l KCl agar bridge was used as a ground.

Evoked action potentials were recorded in current-clamp mode by injecting constant current to maintain the cell at -70mV and then applying 50 or 500 ms steps of increasing current (∆2-7 pA). Spontaneous activity was recorded without current injection in bath solution containing either 1 mmol/l or 10 mmol/l glucose. Data were analysed using Clampfit version 10.7.03 (Molecular Devices) or patchmaster version 2x90.1 (HEKA) software and plotted using Graphpad Prism (version 9, GraphPad Software, USA)..

**References for supplementary methods**


[1] Miedzybrodzka EL, Foreman RE, Galvin SG, et al. (2020) Organoid Sample Preparation and Extraction for LC-MS Peptidomics. STAR Protoc 1(3): 100164. 10.1016/j.xpro.2020.100164

[2] Martin M (2011) Cutadapt removes adapter sequences from high-throughput sequencing reads. EMBnetjournal 17: 10-12. https://doi.org/10.14806/ej.17.1.200.

[3] Laros JFJ (2023) Demultiplex: FASTA/FASTQ demultiplexer. . Available from https://doi.org/10.5281/zenodo.8362959

[4] Dobin A, Davis CA, Schlesinger F, et al. (2013) STAR: ultrafast universal RNA-seq aligner. Bioinformatics 29(1): 15-21. 10.1093/bioinformatics/bts635

[5] Liao Y, Smyth GK, Shi W (2014) featureCounts: an efficient general purpose program for assigning sequence reads to genomic features. Bioinformatics 30(7): 923-930. 10.1093/bioinformatics/btt656

[6] Andrews S (2010) FastQC: A Quality Control Tool for High Throughput Sequence http://www.bioinformatics.babraham.ac.uk/projects/fastqc/

[7] Love MI, Huber W, Anders S (2014) Moderated estimation of fold change and dispersion for RNA-seq data with DESeq2. Genome Biol 15(12): 550. 10.1186/s13059-014-0550-8

[8] Durinck S, Spellman PT, Birney E, Huber W (2009) Mapping identifiers for the integration of genomic datasets with the R/Bioconductor package biomaRt. Nat Protoc 4(8): 1184-1191. 10.1038/nprot.2009.97

[9] Durinck S, Moreau Y, Kasprzyk A, et al. (2005) BioMart and Bioconductor: a powerful link between biological databases and microarray data analysis. Bioinformatics 21(16): 3439-3440. 10.1093/bioinformatics/bti525
[10] Young MD, Wakefield MJ, Smyth GK, Oshlack A (2010) Gene ontology analysis for RNA-seq: accounting for selection bias. Genome Biol 11(2): R14. 10.1186/gb-2010-11-2-r14
[11] Kay RG, Galvin S, Larraufie P, Reimann F, Gribble FM (2017) Liquid chromatography/mass spectrometry based detection and semi-quantitative analysis of INSL5 in human and murine tissues. Rapid Commun Mass Spectrom 31(23): 1963-1973. 10.1002/rcm.7978
[12] Brighton CA, Rievaj J, Kuhre RE, et al. (2015) Bile Acids Trigger GLP-1 Release Predominantly by Accessing Basolaterally Located G Protein-Coupled Bile Acid Receptors. Endocrinology 156(11): 3961-3970. 10.1210/en.2015-1321
[13] Goldspink DA, Lu VB, Miedzybrodzka EL, et al. (2020) Labeling and Characterization of Human GLP-1-Secreting L-cells in Primary Ileal Organoid Culture. Cell Rep 31(13): 107833. 10.1016/j.celrep.2020.107833
[14] Miedzybrodzka EL, Foreman RE, Lu VB, et al. (2021) Stimulation of motilin secretion by bile, free fatty acids, and acidification in human duodenal organoids. Mol Metab 54: 101356. 10.1016/j.molmet.2021.101356
[15] Friedlander RS, Moss CE, Mace J, et al. (2011) Role of phosphodiesterase and adenylate cyclase isozymes in murine colonic glucagon-like peptide 1 secreting cells. Br J Pharmacol 163(2): 261-271. 10.1111/j.1476-5381.2010.01107.x
